# Inpatient cardiac rehabilitation after implantation of a total artificial heart (Aeson device CARMAT) in case of ventricular septal defect after infarction

**DOI:** 10.1093/ehjcr/ytaf478

**Published:** 2025-09-19

**Authors:** Raluca Fiebiger, Alexey Dashkevich, Michal Nozdrzykowski, Joanna Jozwiak-Nozdrzykowska, Erind Gjermeni

**Affiliations:** Clinic for Cardiology, Median Rehabilitation Center Schmannewitz, Waldstr. 4, 04774 Schmannewitz, Germany; Clinic for Cardiac Surgery, Heart Center Leipzig, Strümpellstr. 39, 04289 Leipzig, Germany; Clinic for Cardiac Surgery, Heart Center Leipzig, Strümpellstr. 39, 04289 Leipzig, Germany; Clinic for Cardiology, Heart Center Leipzig, Strümpellstr. 39, 04289 Leipzig, Germany; Clinic for Cardiology, Median Rehabilitation Center Schmannewitz, Waldstr. 4, 04774 Schmannewitz, Germany

**Keywords:** Case report, Ventricular septal rupture (VSR), Myocardial infarction (MI), Total artificial heart (TAH), AESON CARMAT, Cardiac rehabilitation

## Abstract

**Background:**

Post-infarction ventricular septal defect (VSD) is a rare but life-threatening complication of myocardial infarction, in severe cases needing heart transplantation. The Aeson Total Artificial Heart (TAH), a bioprosthetic device designed to replace both ventricles, offers an alternative for patients unsuitable for conventional therapies.

**Case summary:**

A 69-year-old male presented in cardiogenic shock following an inferior wall ST-elevation myocardial infarction complicated by a large VSD. Initial support included extracorporeal life system. Surgical repair was unfeasible due to the defect’s size and proximity to the atrioventricular valve, leaving insufficient rim for septal reconstruction. Total artificial heart implantation was the only viable option, used as bridge-to-decision therapy given the patient’s advanced age. Post-operative recovery was prolonged, but the patient was successfully transferred to an inpatient rehabilitation facility, where structured physiotherapy, endurance, resistance, and mobility training led to significant functional improvement. Close collaboration with a mechanical circulatory support perfusionist resolved recurring TAH alarms related to intraventricular pressure and communication issues adapting the diuretic and antihypertensive medication. The patient was discharged home in stable condition, achieving substantial physical recovery and independence in managing the device.

**Discussion:**

The Aeson TAH proved to be a safe and effective therapy, in particular, as bridge-to-decision therapy in this complex case of post-infarction VSD. Inpatient cardiac rehabilitation played a pivotal role in optimizing physical recovery, managing device-related challenges, and preparing the early transition to an independent living. This case highlights the potential of advanced bioprosthetic solutions and the benefits of a structured rehabilitation system in managing severe cardiac conditions. Further research is needed to evaluate the long-term outcomes and broader applicability of the Aeson TAH.

Learning points

- The Aeson Total Artificial Heart (TAH) can provide life-saving support and facilitate recovery in patients with ventricular septal defect (VSD).
- A structured rehabilitation programme, including endurance, strength, and mobility training, significantly enhances physical capacity and quality of life. The same protocols used for VAD patients can also be applied to TAH patients.
- Close collaboration with a mechanical circulatory support perfusionist and continuous live monitoring are essential for the effective management of patients with TAH, enabling timely and individualized therapeutic decisions.

## Introduction

Post myocardial infarction (MI), ventricular septal defect (VSD) is a rare but severe complication that typically occurs within 2–8 days following MI and is associated with high mortality.^1^ Despite the decline in incidence to below 1% due to advancements in reperfusion therapies,^2^ VSD remains a clinical challenge.

Post-MI, VSD management relies on surgical repair, with timing being crucial: early surgery (<7 days post-MI) has higher mortality, while delayed surgery allows myocardial stabilization.^3^ Less invasive options like percutaneous closure devices show promise but need further evaluation.^4^ In case of unsuitable anatomy near the atrioventricular valve or large VSD, the only treatment option is heart transplantation, but donor shortages and long waiting periods limit feasibility. Durable mechanical circulatory support (MCS) devices, such as ventricular assist devices (VADs), are typically unsuitable for post-infarction VSD. Extracorporeal biventricular support is an alternative but involves high complication rates, poor quality of life, and prolonged intensive care unit stays.^5^

The Aeson Total Artificial Heart (TAH), introduced in 2021, offers a new bridge-to-transplant option for biventricular heart failure and VSD. On September 2023, a case in Kiel, Germany, reported its first successful use in severe post-infarction VSD, followed by transplantation 4 weeks later.^5^

**Figure 1:**
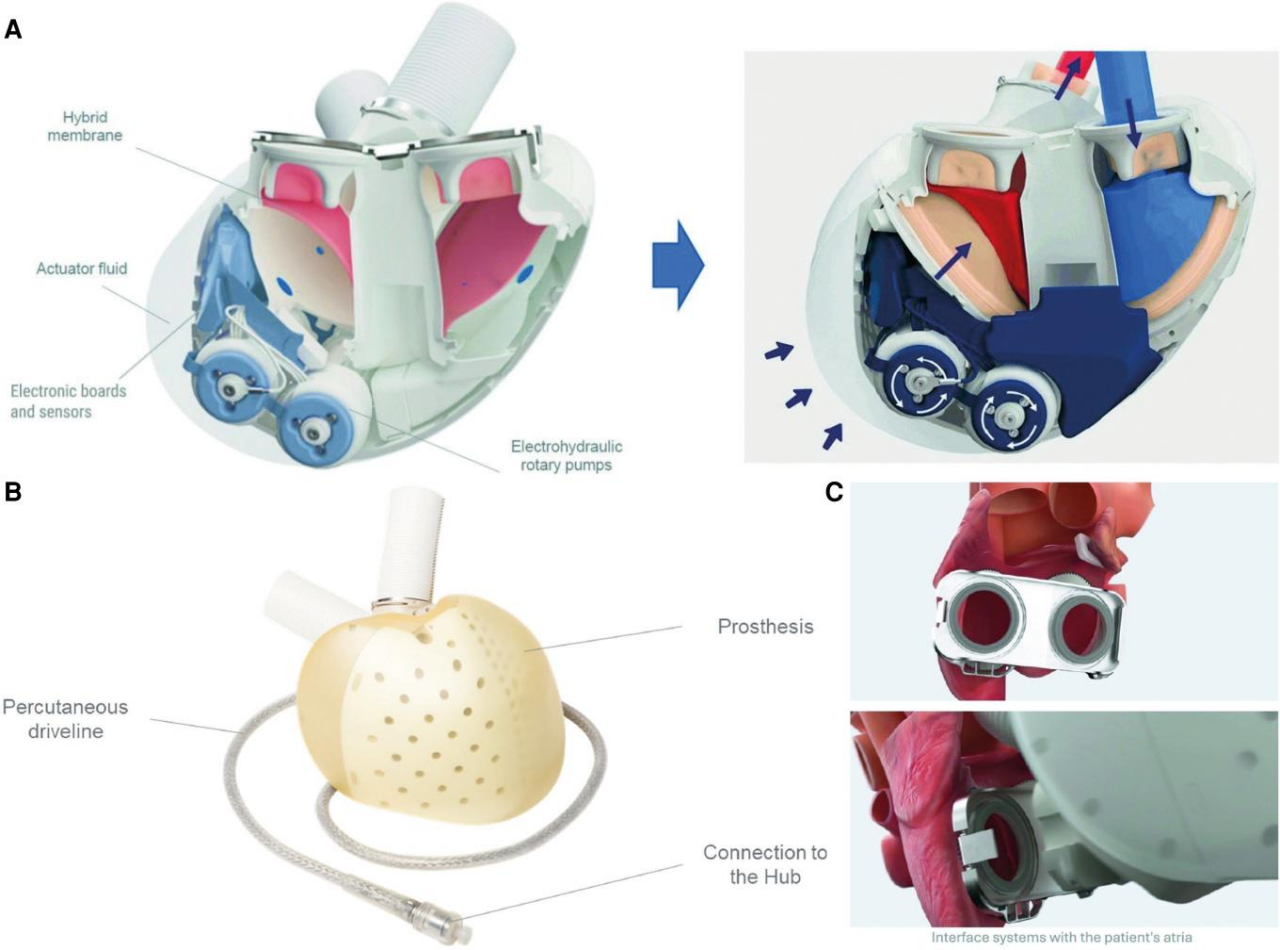
Description of Aeson® components, adapted from the official clinician manual and the ‘2022 Universal Registration Document’.^6,7^ Images reproduced with permission from CARMAT SA. (*A*) Schematic representation of the functional mechanism of the Aeson Total Artificial Heart. (*B*) Illustration of the Aeson Total Artificial Heart system, including the external driveline. (*C*) Visualization of the surgical connection between the Aeson Total Artificial Heart and the atria.

The Aeson TAH (*Figure 1A–C*), a bioprosthetic heart replacing both ventricles, mimics natural heart function with an automated system that continuously adjusts blood flow and heart rate based on the patient's haemodynamic status, reducing complications like thromboembolism and pump dysfunction. The Aeson TAH suits patients needing dual ventricular support or unable to use VADs.^8^ In the future, the company's ambition is to make Aeson TAH the first alternative to a heart transplant.^9^

Here, we report the use of the Aeson TAH as bridge-to-decision in a patient with a large post-infarct VSD, focusing mainly on the experience during stationary cardiac rehabilitation.

## Summary figure

**Table:** 

Date	Event	Details
1 October 2024	ECMO implantation	Due to VSD with cardiogenic shock following an inferior wall MI
7 October 2024	TAH implantation	Performed as a bridge-to-decision therapy after failed ECMO weaning and presence of a large VSD
5 December 2024	Begin inpatient cardiac rehabilitation	Focused on improving mobility, cardiopulmonary capacity, and regaining independence
23 December 2024	Discharge at home	Patient stabilized with significant progress in rehabilitation

## Case presentation

A 69-year-old male with a history of arterial hypertension, hyperlipidaemia, and a positive family history of cardiac disease was referred to a Heart Center on 1 October 2024 in cardiogenic shock caused by a VSD after an inferior wall ST-elevation MI and severe three-vessel coronary artery disease on the same day. Prior to his transfer, he underwent coronary interventions on the right and left anterior descending coronary artery at an external hospital. Despite revascularization, the patient developed a large VSD, resulting in cardiogenic shock that necessitated transfer to the Heart Center. Upon arrival, he was placed on extracorporeal membrane oxygenation (ECMO) support. Computed tomography imaging revealed a large VSD in the basal inferoseptal wall measuring 27 × 22 mm along with mild thinning of the left ventricular apex and suspected apical thrombus measuring 18 × 12 mm, which was later confirmed.

**Figure 2:**
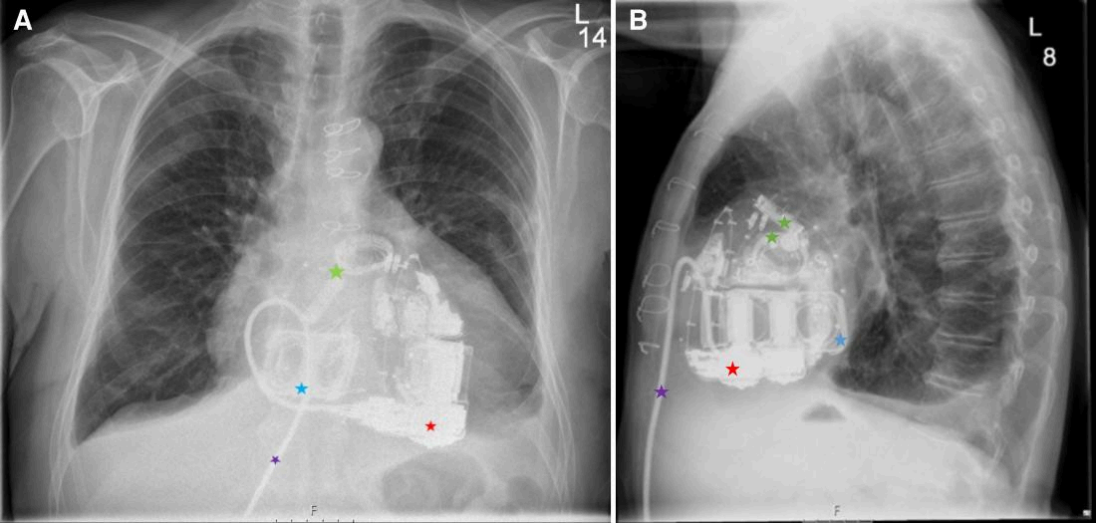
Chest X-ray showing the Aeson® CARMAT Total Artificial Heart, the red star marks the main part of the prosthesis, which contains two micropumps, embedded electronics, and sensors that enable the device to mimic natural heart function through auto-regulated, pulsatile blood flow. The blue star indicates the connection between the atrial flanges and the atrial interface following ventricular removal. The green star marks the pulmonary and aortic valves. The purple star denotes the driveline.

**Figure 3:**
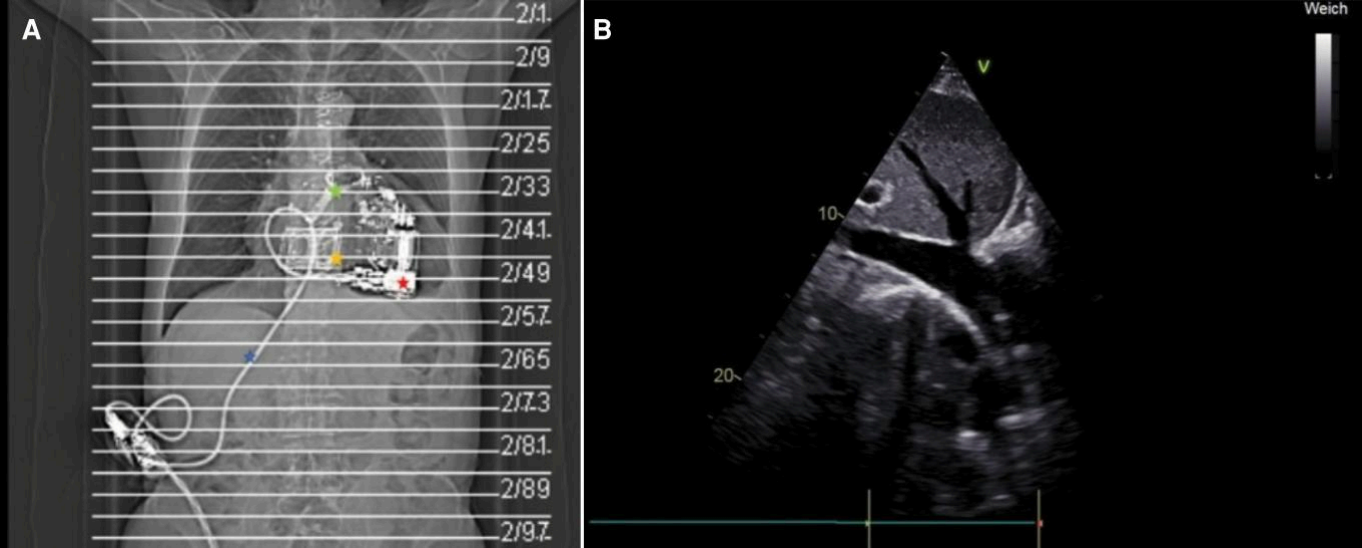
(*A*) Computed tomography of the Aeson® CARMAT Total Artificial Heart device. The red star marks the main part of the prosthesis, which contains two micropumps, embedded electronics, and sensors. The yellow star indicates the connection between the atrial flanges and the atrial interface following ventricular removal. The green star marks the pulmonary and aortic valves. The blue star denotes the driveline. (*B*) Due to acoustic shadowing from the prosthesis, transthoracic echocardiography is limited, allowing only assessment of the inferior vena cava to estimate volume status.

Due to the impossibility of ECMO weaning, the absence of alternative treatment options, and the patient's advanced age, which precluded listing for heart transplantation, an Aeson TAH (*Figure 2*) was implanted as a bridge-to-decision therapy on 7 October 2024, with no intra-operative complications. Post-operative imaging was limited to computed tomography (*Figure 3A*) in cases of suspected pericardial effusion or infection. Due to prosthesis shadowing, transthoracic echocardiography could not assess volume status, which was evaluated via the inferior vena cava (*Figure 3B*). The recovery was prolonged, involving transfusions, temporary dialysis for acute kidney injury, and COVID-19 treated with remdesivir. The anticoagulation was carried out with aspirin 100 mg and tinzaparin 12,000 IU (175 IU/kg) daily. Additionally, the patient received candesartan 8 mg and torasemide 20 mg twice daily and spironolactone 25 mg once daily.

On 5 December 2024, the patient was transferred to our inpatient rehabilitation facility. At admission, he reported severely reduced physical capacity, including impaired fine motor skills, dizziness upon bending, and mild bilateral lower limb oedema. Under TAH support, he maintained low-normal blood pressure, walked short distances with a walker, and denied dyspnoea.

The patient was included in typical cardiac rehabilitation programme over a total of 18 days focused on improving physical capacity, mobility, and education. An interdisciplinary team, including a nutritionist, physiotherapist, and occupational therapist as well as medical personnel, oversaw the programme. Therapy included endurance, resistance/strength, and mobility training. Endurance training included initially cycle ergometer training over 15 min at a pace guided by a subjective Borg index 12–13/20 and inside walking with a walker over 17–20 min. As the patient's condition stabilized, the ergometer training was changed to low-intensity interval training (LIIT), designed to maximize control over patient's workload.^10^ Low-intensity interval training is guided by the perceived intensity of exertion on maximal exercise parameters during an incremental ergometer test (steep ramp). The hard and recovery intervals alternate at 30 s and 60 s, respectively, with the hard interval performed at 50% of the power achieved during steep ramp and recovery intervals with <20 W. Strength training over 15–20 min took place two to three times/week and included low-intensity exercises using machines and elastic bands for upper and lower body muscles. To improve stability and mobility, the patient underwent initially individual training performed in the patient's room and later gymnastic sessions as group training three times/week. The initial programme of 21 days was shortened at patient's wish to spend Christmas at home. During this programme, the patient underwent 19 sessions of endurance training, 6 of strength training, 4 individual trainings, and 7 gymnastics sessions. The programme was complemented by passive physical and balneological treatments aimed at autonomic stabilization.

Finally, his workload capacity increased from an initial constant ergometer workload of 15 W to LIIT 68/15 W, 137 W during a steep ramp test, and 280 m in a 6-min walk test.

**Figure 4:**
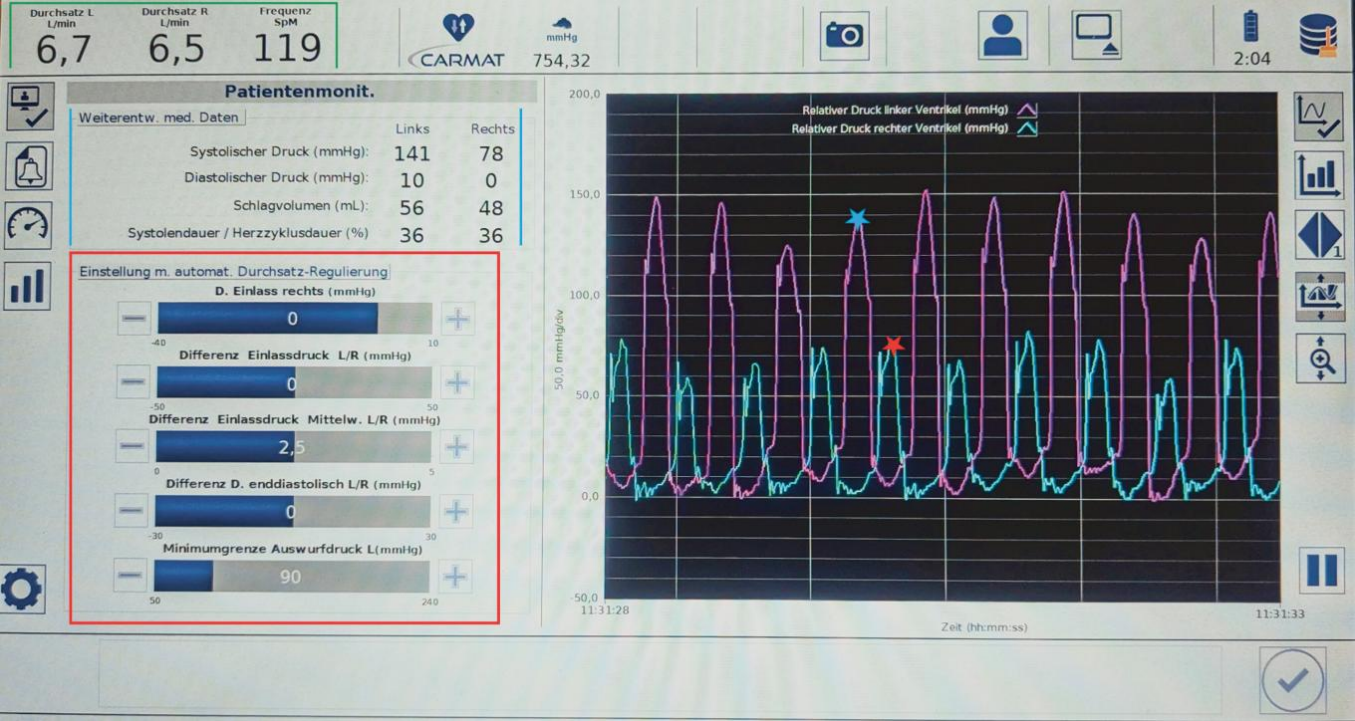
Screenshot of the Aeson® Total Artificial Heart monitor display showing real-time data on pressures and flows. The green box displays the average flow in the left and right chambers, along with the heart rate. Between the blue lines, systolic and diastolic pressures, as well as stroke volumes for both chambers, are shown. The red box highlights the five prosthesis settings available in automatic mode, including inflow and outflow pressures. On the right side, the blue star marks the left ventricular pressure, while the red star indicates the right ventricular pressure.

During inpatient rehabilitation, frequent TAH alarms were triggered due to low pressures in both ventricles caused by reduced preload. Although the patient was asymptomatic with stable vitals, nightly alarms (three to six times) disrupted sleep and recovery. The patient documented the alarm codes, which were reviewed immediately with the MCS perfusionist. As they were non-emergent, alarms were muted at night, and the cause—negative pressure in the right ventricle—was identified via device interrogation (*Figure 4*) on the following day. Despite typically higher preload when lying down, alarms occurred more often in this position for unclear reasons. Discontinuation of diuretics resolved the issue. Later alarms, linked to elevated left ventricular pressure and systolic blood pressure ≥ 180 mmHg, led to adjustments in antihypertensive therapy (increased candesartan, added amlodipine). Throughout, there was close collaboration with the MCS perfusionist from Heart Center.

During the day, the patient was able to participate in therapies without any complaints and showed a significant improvement in his exercise capacity. Managing TAH alarms outside the acute care unit was challenging, but with patient education and collaboration with the MCS perfusionist, the alarms were resolved. The patient was sent home for Christmas in stable condition without alarms, and home care service was arranged for driveline dressing changes.

The patient will undergo close follow-up at the heart centre. During the next appointment, a potential adjustment to an intermediate prophylactic anticoagulation dose (6000–8000 IU/day) will be assessed as part of the long-term management plan, provided that coagulation markers such as D-dimer show a sustained decrease. Aspirin therapy will be continued.

## Discussion

The successful implantation of the Aeson TAH in this patient highlights its potential as a bridge to transplant or destination therapy for severe post-infarction VSD. This case underscores the challenges in managing such complex cases, including multidisciplinary treatment, rehabilitation, and addressing device-related issues. Cardiac rehabilitation played an important role in improving the patient's physical capacity and quality of life, demonstrating the importance of structured rehabilitation programmes in recovery.

The German inpatient rehabilitation system is particularly noteworthy, providing a structured environment that enables early discharge from hospitals while ensuring continuity of care. This model helps alleviate strain on healthcare facilities and promotes patient recovery during extended rehabilitation periods.

A recent case from France also reported the advantage of cardiac rehabilitation in a patient with hypertrophic cardiomyopathy, who developed dilated cardiomyopathy with recurrent cardiac decompensations and cardiogenic shock, implanted with TAH before heart transplantation, with improvement in functional capacity and quality of life.^11^

In conclusion, the Aeson TAH represents a promising solution for patients with complex cardiac conditions, such as large VSDs, where traditional repair or transplantation options are unfeasible.

## Lead author biography


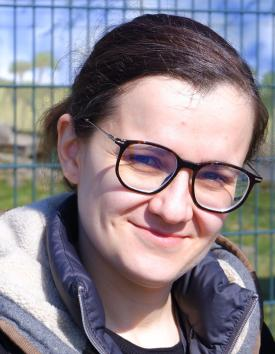


Doctor Raluca Fiebiger is a senior physician at Median Rehabilitation Center Schmannewitz, Germany, since February 2023. She completed her medical studies at the University of Leipzig and became a board-certified specialist in Internal Medicine and Cardiology in June 2020. Her primary interest lies in heart failure, and in 2023, she obtained the Heart Failure Certification from the European Society of Cardiology (ESC). Doctor Raluca Fiebiger is dedicated to improving patient outcomes through evidence-based rehabilitation and cardiovascular care, with a strong focus on heart failure management and advanced cardiology treatments.

**Consent:** Written informed consent was obtained from the patient for the publication of this case report.

## Data Availability

The data underlying this article will be shared on reasonable request to the corresponding author.
